# Genetic Diagnosis Using Whole Exome Sequencing in Common Variable Immunodeficiency

**DOI:** 10.3389/fimmu.2016.00220

**Published:** 2016-06-13

**Authors:** Patrick Maffucci, Charles A. Filion, Bertrand Boisson, Yuval Itan, Lei Shang, Jean-Laurent Casanova, Charlotte Cunningham-Rundles

**Affiliations:** ^1^Immunology Institute, Icahn School of Medicine at Mount Sinai, New York, NY, USA; ^2^Division of Clinical Immunology, Department of Medicine, Icahn School of Medicine at Mount Sinai, New York, NY, USA; ^3^Rockefeller Branch, St. Giles Laboratory of Human Genetics of Infectious Diseases, The Rockefeller University, New York, NY, USA; ^4^Necker Branch, Laboratory of Human Genetics of Infectious Diseases, INSERM U1163, Necker Hospital for Sick Children, Paris, France; ^5^Imagine Institute, Paris Descartes University, Paris, France; ^6^Howard Hughes Medical Institute, New York, NY, USA; ^7^Pediatric Hematology-Immunology Unit, Necker Hospital for Sick Children, Paris, France

**Keywords:** common variable immunodeficiency, whole exome sequencing, next-generation sequencing, primary immunodeficiencies, genetic diagnosis

## Abstract

Whole exome sequencing (WES) has proven an effective tool for the discovery of genetic defects in patients with primary immunodeficiencies (PIDs). However, success in dissecting the genetic etiology of common variable immunodeficiency (CVID) has been limited. We outline a practical framework for using WES to identify causative genetic defects in these subjects. WES was performed on 50 subjects diagnosed with CVID who had at least one of the following criteria: early onset, autoimmune/inflammatory manifestations, low B lymphocytes, and/or familial history of hypogammaglobulinemia. Following alignment and variant calling, exomes were screened for mutations in 269 PID-causing genes. Variants were filtered based on the mode of inheritance and reported frequency in the general population. Each variant was assessed by study of familial segregation and computational predictions of deleteriousness. Out of 433 variations in PID-associated genes, we identified 17 probable disease-causing mutations in 15 patients (30%). These variations were rare or private and included monoallelic mutations in *NFKB1*, *STAT3*, *CTLA4*, *PIK3CD*, and *IKZF1*, and biallelic mutations in *LRBA* and *STXBP2*. Forty-two other damaging variants were found but were not considered likely disease-causing based on the mode of inheritance and/or patient phenotype. WES combined with analysis of PID-associated genes is a cost-effective approach to identify disease-causing mutations in CVID patients with severe phenotypes and was successful in 30% of our cohort. As targeted therapeutics are becoming the mainstay of treatment for non-infectious manifestations in CVID, this approach will improve management of patients with more severe phenotypes.

## Introduction

Common variable immunodeficiency (CVID) is the most common symptomatic group of known primary immunodeficiency (PID) syndromes, affecting approximately 1 in 25,000 people (1). This diagnosis is characterized by decreased IgG and either low IgA or IgM, absent or deficient specific antibody responses to infection or vaccination, and exclusion of other causes of hypogammaglobulinemia (1). Patients with CVID commonly have a history of sinopulmonary infections, but over half experience other complications, including autoimmunity, interstitial lung disease, lymphoid hyperplasia, inflammatory bowel disease, nodular regenerative hyperplasia of the liver, granulomatous infiltrations, or malignancy (2). The clinical heterogeneity, combined with a variable age of onset, suggests that this syndrome is a collection of clinical entities caused by a number of distinct genetic defects. Genetic analyses of subjects with a CVID phenotype have identified recessively inherited traits with biallelic mutations in *ICOS*, *CD19*, *CD20*, *CD21*, *CD81*, *PRKCD*, and *LRBA*, and autosomal dominant traits with monoallelic mutations in *PIK3CD*, *NFKB2*, *PIK3R1* (1), and, most recently, *NFKB1* (3) and, as we have shown, *IKZF1* (4). In addition, hypomorphic mutations in genes whose null mutations are associated with severe combined immune deficiency have been identified in rare CVID cases (5), illustrating the broad phenotypic spectrum of mutations at these loci.

Whole exome sequencing (WES) has proven an effective tool for the discovery of mutations in novel PID-causing genes in patients with syndromes of unknown etiology (6). WES can also help reach a diagnosis in unknown genetic disorders. A recent observational study of exome-sequenced patients with suspected genetic disorders reported a molecular diagnosis rate of approximately 25% (7). In this approach, sequenced exomes are aligned with reference genomes, allowing identification of disease-related mutations. Candidate variations are then screened through databases that provide information on allele frequency, allowing elimination of mutations that do not match disease occurrence or the predicted method of inheritance. Despite the success of next-generation sequencing (NGS), the best approach for genetic diagnosis in patients with the CVID syndrome has been unclear since selection of patients, insurance reimbursement policies, and, most importantly, the complexity of variant analysis are obvious hurdles. The goal of this study is to outline a practical approach for clinicians seeking to diagnose genetic defects leading to more severe CVID phenotypes. We report here the findings of WES of 50 selected CVID patients combined with a targeted screening approach to identify pathogenic mutations in genes known to cause PIDs.

## Materials and Methods

### Patient Selection

Subjects were diagnosed with CVID using established criteria, including serum IgG and IgA and/or IgM deficiency with proven loss of antibody production (1) and enrolled in a Mount Sinai institutional review board-approved protocol for this study. The subjects selected for WES met one or more of the following criteria: early-onset of manifestations (under age 10) (36%), autoimmune/inflammatory manifestations (76%), low B lymphocyte counts (58%), and/or familial history of hypogammaglobulinemia (16%). Male subjects with known causes of absent B cells (X-linked agammaglobulinemia) were excluded. In contrast to previous reports, which have described autosomal recessive variants, no subjects in this report had a background suggestive of consanguinity. When available, samples from parents and siblings of CVID patients were submitted for whole exome and/or Sanger sequencing to study familial segregation.

### Whole Exome Sequencing

Genomic DNA was extracted from peripheral blood mononuclear cells and sheared with a Covaris S2 Ultrasonicator. An adaptor-ligated library was prepared with the Paired-End Sample Prep kit V1 (Illumina). Exome capture was performed with the SureSelect Human All Exon kit (Agilent Technologies). Massively parallel sequencing was performed on a HiSeq 2500 (Illumina), which generates 100-base reads. Sequences were aligned for variant calling and annotation with the human genome reference sequence (hg19 build) using BWA aligner (8). Downstream processing was performed with the genome analysis toolkit (GATK) (9), SAMtools (10), and Picard Tools (http://picard.sourceforge.net/). A GATK UnifiedGenotyper and a GATK IndelGenotyperV2 were used to identify substitution and indel variant calls, respectively. Calls with a read coverage of ≤2× and a Phred-scaled SNP quality of ≤20 were filtered out. All variants were annotated with the GATK Genomic Annotator (Broad Institute).

### Targeted Gene Screening

Patient exomes were filtered for mutations in 269 genes associated with PIDs (Table S1 in Supplemental Material). Heterozygous and homozygous mutations were excluded if the allele frequencies in the general population were >0.01 and 1.0%, respectively, in the Exome Aggregation Consortium database (ExAC, Broad Institute). Top likely disease-causing candidates were Sanger sequenced for confirmation (primers in Table S2 in Supplemental Material). Familial segregation was studied when samples were available. Other candidate mutations were confirmed by examining read alignment in the integrated genomics viewer (IGV; Broad Institute). All confirmed mutations were subsequently analyzed using computational predictors of mutation severity, including combined annotation-dependent depletion (CADD) (11), and were compared with the gene-specific mutation significance cutoff (MSC) (12). Variants with CADD scores below the gene-specific MSC were excluded. Confirmed variations were also screened through the Human Gene Mutation Database (13) to identify published disease-associated variations.

## Results

**Figure 1:**
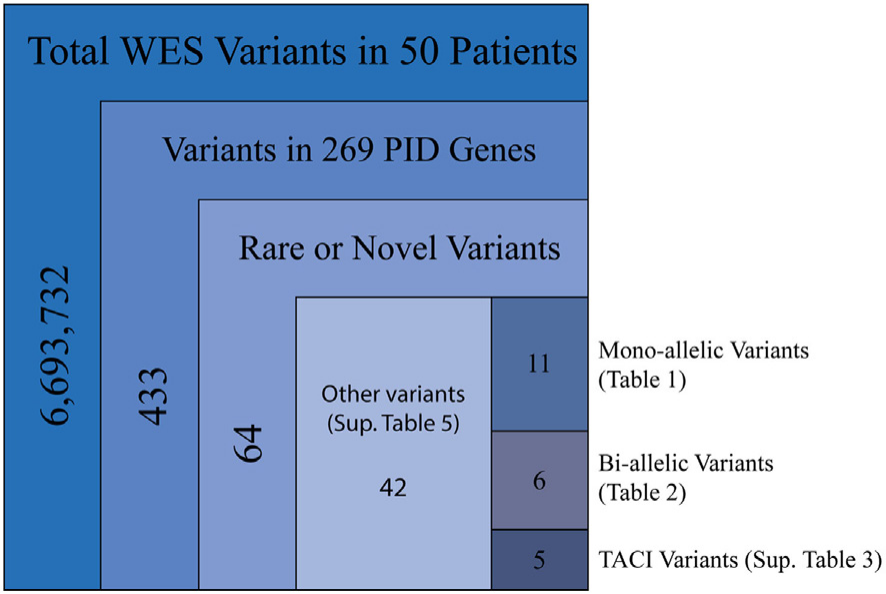
**Filtering strategy for WES and the 269 PID gene screen**. Includes sequencing data for all 50 patients, of which 38 were found to have mutations in PID-associated genes. Novel variants were those not reported in ExAC, whereas rare variants were reported with a frequency of <0.1% (heterozygous) or 1.0% (homozygous).

**Figure 2:**
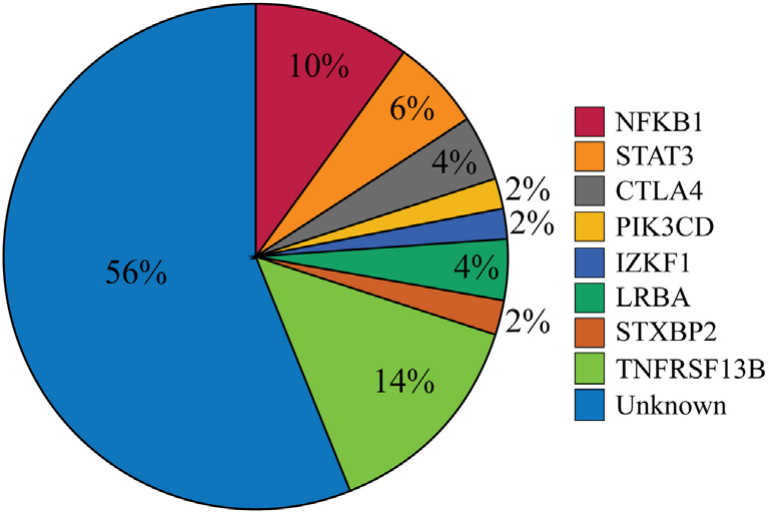
**Percentages of patients with likely disease-causing or -associated mutations**. One patient had mutations in both *PIK3CD* and *TNFRSF13B* but was only included in the *PIK3CD* category. The Unknown category contains both patients for whom no variations in PID-associated genes were found (*n* = 12) and those who were found only to have a mutation reported in Table S5 in Supplementary Material (*n* = 16).

Our cohort of 50 patients included 42 sporadic and 8 familial cases. The familial cases included two sisters, two brothers, father and son, and two second cousins. The average age of patients was 36 years, and 54% of patients were females. Following sequence alignment and variant calling, patient exomes were filtered to identify significant mutations in 1 or more of 269 immune deficiency-related genes (Table S1 in Supplemental Material). This approach revealed 433 variations in 38 patients (76% of the cohort), of which 64 (in 38 genes) were either private or rare (Figure 1). Seventy-two percent of these variations are estimated to be in the top 1% of all human hg19 reference SNVs (CADD-scaled score ≥20) and 20% in the top 0.1% (CADD-scaled score ≥30). Eleven have been published as disease-causing or -associated. Of the 64 private or rare variations identified, 17 (Tables 1 and 2) were considered likely disease-causing in 15 patients (30%) (Figure 2; Tables 3 and 4). We also noted 5 variants in 8 patients in *TNFRSF13B* (Tables S3 and S4 in Supplementary Material), which is known to be weakly associated with CVID (14), and 42 variants in other genes associated with PIDs (Tables S5 and S6 in Supplementary Material).

**Table 1:** **Monoallelic mutations**.

Patient	Gene	Refseq transcript	Coding change	Protein change	CADD	ExAC freq
1	*NFKB1*	NM_003998.3	c.1301-1G > A		24	–
2			c.1301-1G > A		24	–
3			c.259-4A > G		9.635	0.000008256^a^
4			c.957T > A	p.Y319*	36	–
5			c.1375delT	p.F459Lfs*26	23.2	–
6	*STAT3*	NM_139276.2	c.737G > A	p.R246Q	33	–
7			c.937T > C	p.F313L	16.66	–
8			c.307C > T	p.R103W	34	–
9	*CTLA4*	NM_005214.4	c.56_57insCTGG	p.T19Tfs*42	19.5	–
10			c.406C > G	p.P136A	23.8	–
11	*PIK3CD*	NM_005026.3	c.3061G > A	p.E1021K^b^ (16)	31	–
12	*IKZF1*	NM_006060.5	c.551G > A	p.R184Q	27.9	–

*^a^Variant reported in a single patient in the ExAC database*.

*^b^Published disease-causing variant*.

**Table 2:** **Biallelic mutations**.

Patient	Gene	Refseq transcript	Coding change	Protein change	CADD	ExAC freq
13	*LRBA*	NM_006726.4	c.1399A > G	p.M467V	19	0.002165
			c.8351C > G	p.A2784G	25.7	–
14			c.2674G > A	p.A892T	24.4	0.001532
			c.6695T > C	p.I2232T	27.9	0.0002160
15	*STXBP2*	NM_006949.3	c.474_483delinsGA	p.C158Wfs*78^a^ (27)	33	–
			c.1001C > T	p.P334L^a^ (27)	23.4	0.00004944

*^a^Published disease-causing variant*.

**Table 3:** **Clinical and immunological phenotypes of patients with monoallelic mutations**.

Patient	Sex	Age/age at onset (years)	Infections	Other conditions	IgG (7.00–16.00 g/L)^a^	IgA (0.70–4.00 g/L)^a^	IgM (0.40–2.30 m/L)^a^	CD3^+^ (750–2500/mm^3^)^a^	CD4^+^ (480–1700/mm^3^)^a^	CD8^+^ (180–1000/mm^3^)^a^	CD3^−^CD56^+^ (135–525/mm^3^)^a^	CD19^+^ (75–375/mm^3^)^a^/CD19^+^CD27^+^IgD^−^%^b^
***NFKB1***												
1^c^	M	Died at 51/42	Pneumonias; chronic sinusitis; conjunctivitis; otitis; shingles	Lung granulomas; enteropathy; lymphoid hyperplasia; neutropenia; hypersplenism (s/p splenectomy); aphthous ulcers	Tx^d^	<0.07 (↓)	<0.11 (↓)	378 (↓)	268 (↓)	117 (↓)	50 (↓)	7 (↓)/0
2^c^	F	33/19	Chronic sinusitis; pneumonias; conjunctivitis; shingles; otitis; *C. difficile* colitis	Morphea	<0.51 (↓)	<0.05 (↓)	<0.05 (↓)	1428	1007	435	53 (↓)	21 (↓)/NA
3	M	48/21	Pneumonias; empyema (s/p lobectomy); chronic sinusitis	Bronchiectasis; vitiligo, hypothyroidism	Tx^d^	<0.01	<0.05 (↓)	682 (↓)	323 (↓)	336	45 (↓)	62 (↓)/0
4	F	Died at 48/19	MAI; *Pneumocystis jirovecii* pneumonia; lung abcesses; PML (cause of death)	ITP (s/p splenectomy); AIHA; aplastic bone marrow	Tx^d^	0.07 (↓)	0.17 (↓)	NA	1177	589	NA	0 (↓)/0
5	F	25/7	Pneumonias, otitis; giardiasis; *C. difficile* colitis; HSV infection; cellulitis; MAI	Bronchiectasis, enteropathy; osteopenia; poor growth	Tx^d^	0.02	0.02 (↓)	473 (↓)	518 (↓)	153 (↓)	98 (↓)	0 (↓)/0.06
**STAT3**												
6	F	55/11	Brain abscess; West Nile virus encephalitis	Liver and lung granulomas; ITP; hepatopulmonary syndrome; Severe arthritis; AIHA (s/p splenectomy), hemorragic stroke; DVT; hypothyroidism	0.60 (↓)	0.07 (↓)	0.20 (↓)	2223	1254	902	170	562 (↑)/0
7	M	18/3	*Molluscum contagiosum*; *Staphylococcus* infections; tinea corporis; *C. difficile* colitis	Type 1 diabetes; ITP/Evans syndrome hypothyroidism; short stature	6.81 (↓)	<0.07 (↓)	0.37	715 (↓)	337 (↓)	276	83 (↓)	401/NA
8	F	18/5	*Molluscum contagiosum*	Microscopic colitis; ITP, granulomatous lung disease; lymphoid hyperplasia; pure red cell aplasia; atopic dermatitis; malabsorption	3.40 (↓)	0.08 (↓)	0.20 (↓)	1180	641	432	92 (↓)	237/NA
**CTLA4**												
9	F	17/1	Conjunctivitis; warts	Inflammatory bowel disease; LIP, ITP; AIHA, autoimmune neutropenia; erythema nodosum; failure to thrive; osteoporosis	5.94 (↓)	<0.05 (↓)	0.08 (↓)	756 (↓)	477	234 (↓)	83 (↓)	93 (↓)/2.51
10	M	24/14	*Molluscum contagiosum*; influenza A; otitis media; recurrent sinusitis, bronchitis, and otitis media	Follicular bronchiolitis; kidney, lung, and lymph node granulomas; ITP/AIHA	3.10 (↓)	0.22 (↓)	3.87 (↑)	877	457	410	140	7 (↓)/0.3
**PIK3CD**												
11	F	16/3	Chronic otitis media; pneumonias; sinusitis; periorbital cellulitis; HSV infection; conjunctivitis; Giardiasis	Chronic colitis with malabsorption and short stature; ILD; nodular hyperplasia; osteoporosis; splenomegaly	5.84 (↓)	<0.11 (↓)	5.87 (↑)	1033	278 (↓)	594	186	388/0.65
**IKZF1**												
12	F	26/9	Recurrent pneumonias		0.42 (↓)	<0.01 (↓)	0.07 (↓)	3388	1416	1888	128 (↓)	<1 (↓)/NA

*AIHA, autoimmune hemolytic anemia; DVT, deep venous thrombosis; HSV, herpes simplex virus; ILD, interstitial lung disease; ITP, immune thrombocytopenic purpura; LIP, lymphocytic interstitial pneumonia; NA, not available; MAI, *Mycobacterium avium intracellulare*; PML, progressive multifocal leukoencephalopathy*.

*^a^Normal value ranges in patients aged six or more*.

*^b^Percentage of total CD19^+^ cells*.

*^c^Patients 1 and 2 are second cousins*.

*^d^Patients were already on IgG replacement therapy when evaluated for the first time in our center*.

**Table 4:** **Clinical and immunological phenotypes of patients with biallelic mutations**.

Patient	Sex	Year of birth/age at onset (years)	Infections	Other conditions	IgG (7.00–16.00 g/L)^a^	IgA (0.70–4.00 g/L)^a^	IgM (0.40–2.30 g/L)^a^	CD3^+^ (750–2500/mm^3^)^a^	CD4^+^ (480–1700/mm^3^)^a^	CD8^+^ (180–1000/mm^3^)^a^	CD3-CD56^+^ (135–525/mm^3^)^a^	CD19^+^ (75–375/mm^3^)^a^/CD19^+^CD27^+^IgD^−^%^b^
***LRBA***												
13	F	Died at 39/25	Cellulitis; pneumonias	Liver and pulmonary granulomas leading to organ failures (cause of death); ITP; splenectomy	5.56 (↓)	<0.07 (↓)	0.17 (↓)	2995	1579	1479	363	4 (↓)/0
14	M	Died at 56/36	Fulminant; mycoplasma infection; recurrent bronchitis	Lung granuloma; AIHA, ITP; squamous-cell carcinoma of the mouth	1.72 (↓)	<0.07 (↓)	0.06 (↓)	764	331 (↓)	411	19 (↓)	0 (↓)/0
***STXBP2***												
15	M	35/28	EBV age 28 with neutropenia; recurrence of EBV at age 34; recurrent sinusitis; shingles ×2	IBD	0.81 (↓)	<0.05 (↓)	<0.05 (↓)	1623	1250	304	32 (↓)	9 (↓)/0

*AIHA, autoimmune hemolytic anemia; EBV, Epstein–Barr virus; IBD, inflammatory bowel disease; ITP, immune thrombocytopenic purpura*.

*^a^Normal value ranges in patients aged six or more*.

*^b^Percentage of total CD19^+^ cells*.

### Monoallelic Mutations

Five patients were identified with mutations in *NFKB1*, which encodes the NF-κB1 p105 subunit that is processed into the active p50 transcription factor (15). Three patients were sporadic cases, while two were second cousins. The mutations in *NFKB1* included two nucleotide substitutions that may affect splicing (c.1301-1G > A in the related patients 1 and 2; c.259-4A > G in patient 3), one nonsense mutation (p.Y319* in patient 4), and one frameshift deletion (p.F459Lfs*26 in patient 5) (Table 1). Three of these variations are novel and none have been previously described as disease-causing. All five patients initially had profound hypogammaglobulinemia and various degrees of autoimmune and inflammatory manifestations, and four patients were diagnosed with opportunistic infections (Table 3). Despite immunoglobulin replacement, two patients (#2 and 4) did not survive.

We also identified novel mutations in *STAT3* (Table 1) in three unrelated patients who had a history of autoimmune cytopenias and granulomatous organ infiltration (Table 3). The variant in patient 6 (p.R246Q) is *de novo*, while the mutation in patient 7 (p.F313L) was inherited from his unaffected father. Patient 8 shares her mutation (p.R103W) with her mother and two siblings, suggesting incomplete penetrance. Two unrelated patients with low B cells and severe autoimmunity had novel mutations in the regulatory receptor *CTLA4* gene. One of these mutations, p.T19Tfs*42, was identified in both patient 9 and her asymptomatic mother, also suggesting incomplete penetrance, while the other, p.P136A, in patient 10 was *de novo*. In one patient (#11) with recurrent sinopulmonary infections and severe pulmonary and gastrointestinal manifestations, we identified the gain-of-function substitution p.E1021K in *PIK3CD*, a gene which codes for p110δ, the catalytic subunit of phosphoinositide 3-kinase δ (PI3Kδ) (16). Finally, we also noted an amino acid substitution (p.R184Q) in patient 12 in *IKZF1*, which codes for the hematopoietic zinc finger transcription factor IKAROS (17). This patient exhibited agammaglobulinemia with the absence of B cells and recurrent pneumonias starting at the age of 9. Her mother, who is mildly hypogammaglobulinemic, shares this mutation, suggesting variable expressivity. Patient 12 is included in a report demonstrating that the p.R184Q mutation and others are deleterious to the ability of IKAROS to bind its consensus sequence and properly localize in the nucleus (4).

### Biallelic Mutations

Two unrelated adult patients with sporadic CVID, granulomatous disease, and autoimmune cytopenias (Table 4) had mutations in *LRBA*, a protein implicated in regulation of cell survival (18), endosomal trafficking (19), and regulation of CTLA4 (20). The four mutations (p.M467V and p.A2784G in patient 13; p.A892T and p.I2232T in patient 14) are rare or novel and have not been previously associated with clinical disease (Table 2). Although the CADD scores for these mutations fall below the MSC for *LRBA*, we include them as the patient phenotypes closely match previously reported cases (19, 21). Sanger sequencing of parents revealed that the patients are compound heterozygous for these variants. Compound heterozygous mutations in *STXBP2* (p.C158Wfs*78 and p.P334L) were also identified in one patient (#15) who had very low B cells and a prior severe EBV infection associated with neutropenia that responded to corticosteroids, cyclosporine, and filgrastim. *STXBP2*, which encodes syntaxin-binding protein 2, has a role in cytotoxic T and NK cell functions and is associated with familial hemophagocytic lymphohistiocytosis (FHL), a clinical phenotype not found in this patient (22). Sanger sequencing indicated that the patient inherited one *STXBP2* mutation from each parent.

### Mutations in TNFRSF13B (TACI)

As in previous reports in CVID, we identified known variations in *TNFRSF13B*, which codes for transmembrane activator and calcium-modulating cyclophilin ligand interactor (TACI). Although some of these variations occur at a frequency greater than our specified cutoff, we included them as validation for our cohort, as *TNFRSF13B* mutations have been identified in approximately 10% of CVID patients (14). These variations occurred in eight patients and include one unreported mutation in a single patient (#11) who also had the p.E1021K substitution in *PIK3CD* (Tables S3 and S4 in Supplementary Material). All *TNFRSF13B* variations were monoallelic except for patient 20, whose variations are biallelic as determined by WES of siblings.

### Other Damaging Mutations

We also identified 42 other heterozygous mutations (Table S5 in Supplementary Material) in our patients (Table S6 in Supplementary Material). A majority of these variants (*n* = 30) have been previously reported in ExAC and, of those, four have already been published as disease-causing (noted in Table S5 in Supplementary Material). In addition, CADD scores for all variants included in Table S5 in Supplementary Material exceed their gene-specific MSC. However, they were not considered to be likely disease-causing in our patients since the deficiency caused by variations in the gene did not match the phenotype of the patient and/or the zygosity of the mutation in the patient did not match the published inheritance pattern for the disease. Of note, we identified two mutations in *DOCK8* (p.R1008Q and p.E1104D) in patient 19. Family studies indicated that both these mutations were inherited from the patient’s father, who is healthy. Though the mutations are predicted to be highly damaging by CADD, the patient demonstrated recurrent sinopulmonary infections and severe granulomatous infiltrations but no cardinal manifestations of *DOCK8* deficiency (23). We also found one variant in *FASLG* (p.R198W) in two sisters (patients 27 and 28) with recurrent pneumonias and low B cells. Interestingly, three unrelated patients (#24, 32, and 33) carry a damaging heterozygous variant (p.R1305H) in *LRBA*. All three patients had recurrent sinopulmonary infections, and two shared notable organomegaly, hepatic nodular regenerative hyperplasia, and autoimmune cytopenias. Copy number variation in these patients has not yet been explored.

## Discussion

While CVID has long been considered a clinically heterogeneous group of PIDs (1), the gene defects that underlie this complex syndrome have been difficult to identify, particularly in non-familial cases. In this study, we collected complete exome data for 50 selected CVID subjects, most of whom were sporadic, who had severe phenotypes found in about half of all CVID cases (2). Our goal was to identify variants leading to these manifestations, filtering first for a panel of causative genes. The filtering results demonstrated likely disease-causing mutations in *NFKB1*, *STAT3*, *CTLA4*, *PIK3CD*, *IKZF1*, *LRBA*, and *STXBP2* in 15 patients (30%) and disease-associated mutations in TNFRSF13B in 8 patients (16%) (Figure 2). Our findings indicate that seeking pathogenic PID mutations in sporadic CVID is a fruitful approach. While eight subjects were familial cases of CVID, the same damaging variant was noted in only two of these subjects (second cousins), illustrating that additional genes leading to familial B cell defects remain unidentified.

Private or rare heterozygous mutations were identified in three transcription factors (*NFKB1*, *STAT3*, and *IKZF1*) and two signaling proteins (*CTLA4* and *PIK3CD*). Mutations in four genes, *NFKB1*, *STAT3*, *CTLA4*, and *PIK3CD*, led to not only profound B cell defects but also significant immune dysregulation, including autoimmunity, lymphoid hyperplasia, and organ infiltrative granulomatous disease, complications described in other subjects with familial inheritance (3, 16, 24, 25). In our patients, mutations in *NFKB1* and *STAT3* also led to opportunistic infections, including *Mycobacterium avium intracellulare*, JC virus-induced progressive multifocal leukoencephalopathy, *Pneumocystis jirovecii* pneumonia, and *Molluscum contagiosum*. In contrast, the *IKZF1* mutation found in one patient led to near agammaglobulinemia and bacterial infections but no evidence of immune dysregulation or susceptibility to additional organisms (4). While two related CVID patients had the same *NFKB1* variant, one healthy family member of those patients and healthy family members of subjects with *STAT3* and *CTLA4* mutations were found to have the same variants, illustrating the variable penetrance of monoallelic variations in these genes, as previously reported (3, 25, 26). However, the *STAT3* variants p.F313L and p.R103W found in patients 7 and 8 possess gain-of-function properties and have been identified in other confirmed cases of early onset autoimmune disease, validating the pathogenic roles of these mutations in our patients (Vogel and Cooper, personal communication).

Compound heterozygous mutations were also identified in three subjects with sporadic disease. Of these, two patients with profound B cell defects, autoimmunity, and granulomatous disease had novel mutations in *LRBA* and died in middle age of organ damage. Homozygous mutations in this gene have previously been associated with severe CVID phenotypes associated with loss of regulatory T-cell functions (19, 21). Here, we found patients with apparently adult-onset CVID and no family history who exhibited a similar severe phenotype. Another patient with hypogammaglobulinemia, inflammatory bowel disease, and a history of EBV infection had compound heterozygous mutations in *STXBP2*. However, he never displayed hemophagocytosis, generally associated with *STXBP2* mutations (22). A previous report described a patient with severe FHL requiring hematopoietic stem cell transplant who had the same two *STXBP2* mutations, indicating that these are likely to be deleterious (27). The reason for the discordance between our patient’s phenotype, with a predominant B cell defect, and the previously reported cases is unknown.

In addition to the genes identified that are associated with loss of antibody function and inflammatory complications, we also identified rare or novel monoallelic variants that, in other patients, are known to cause different immune deficiency syndromes. These include the p.W688* mutation in *CIITA* in patient 3, previously reported in a patient with a compound heterozygous MHC Class II deficiency (28), and the p.R1445Q substitution in *LRBA* in patient 2, which led to loss of CTLA4 and immune dysregulation in a patient when biallelic (20). Curiously, we also noted a p.R1305H substitution in *LRBA* in three other unrelated patients. The impact of such heterozygous mutations on immune functions in these and similar subjects is unclear.

Using whole genome sequencing, van Schouwenburg et al. recently published results for 31 sporadic CVID patients in which they identified 112 variants (38 novel) in pathways that could lead to CVID-like antibody failure (29). In contrast to our study, the CVID patients examined in this report were not pre-selected based on the severity of clinical and/or immunological phenotypes. When applying our filtering criteria, none of the variants described in their study would be considered likely causative of CVID. In our study, pre-selection of subjects with more severe non-infectious complications most likely contributed to identification of novel and rare pathogenic mutations.

Family studies in cohorts have been enormously valuable for exploring the pathogenesis of CVID, revealing many genes essential for B cell development and maintenance (3, 4, 26, 30). However, the utility of sequencing single genes or a panel of genes in patient populations is limited and not cost-effective. For example, in 699 PID subjects, mutations in *PIK3CD* were identified in only 3 siblings with CVID (31). Sequencing panels that are commercially available provide an important and indispensable alternative to clinicians. Such panels are designed with probes that target genes relevant for specific diseases or phenotypes and may provide higher coverage and sequencing depth than WES. These panels are often appropriate for the genetic diagnosis of PIDs presenting with clear-cut clinical and/or immunological phenotypes but, if emerging causative genes are not yet incorporated, subjects with CVID, who may have heterogeneous phenotypes, will not be diagnosed. Furthermore, gene panels could be more expensive than WES are not as easily updated and, in contrast to WES, require repeat testing of patient samples as new PID-associated genes are described. Combining WES with an expandable PID-associated gene filter streamlines the search for disease-causing variants. The success of NGS also guides physicians toward therapeutic approaches that target the deficient or dysregulated protein in a patient. Notable examples include utilizing tocilizumab in subjects with *STAT3* gain-of-function variants (26), CTLA-4-Ig fusion protein in subjects with *CTLA4* haploinsufficiency or *LRBA* deficiency (20, 32), and rapamycin, which inhibits mTOR downstream of PI3K, in patients with gain-of-function *PIK3CD* mutations (33). This approach may be of considerable value in CVID subjects with more severe phenotypes, such as those examined here.

The use of NGS to identify mutations associated with immunodeficiency diseases has become increasingly common. In a recent 5-year period, NGS identified more than 30 new molecular etiologies of PIDs (6). Our results also indicate the higher yield of WES when targeting CVID patients with severe clinical or immunological complications, even those with no family history of immune deficiency. With the pronounced overlap between the inflammatory conditions found in CVID, WES followed by periodic filtering with an expanding panel of genes may provide a practical means for genetic diagnosis of these subjects. However, while costs of NGS have dropped substantially in recent years, price is still a barrier. Equally important, for most subjects with CVID, the relevant genes have not been identified, leading to continued effort to search for novel genes contributing to the CVID phenotype. This is currently ongoing at this and other centers.

## Consent

Proper consent has been obtained from all patients or from the parents of minors included in this study.

## Author Contributions

PM, BB, J-LC, and CC-R conceived and designed the study. PM analyzed and managed data, and created figures. PM and BB interpreted data. CF assembled phenotypic data for study participants. PM and CF performed literature review, created tables, and performed Sanger sequencing of likely disease-causing variants. YI, LS, and BB processed raw WES sequencing data and created filtering method. CC-R recruited patients to the study. PM, CF, and CC-R wrote and prepared the manuscript. All authors read and approved the final manuscript draft before submission.

## Conflict of Interest Statement

PM, BB, YI, LS, J-LC, and CC-R received grants from the National Institutes of Health. CF received financial support from the University of Montreal and the Maisonneuve-Rosemont Hospital. CC-R received support from the Jeffrey Modell Foundation and departmental funding from the David S. Gottesman Chair. J-LC has received grants from Biogen and Merck, and personal fees from Genentech, Sanofi, Novartis, Pfizer, Bioaster, Regeneron, Biogen Idec, and ADMA.

## Abbreviations

CADD, combined annotation-dependent depletion; CVID, common variable immunodeficiency; MSC, mutation significance cutoff; NGS, next-generation sequencing; WES, whole exome sequencing; WGS, whole genome sequencing.
