# Supplementary material for: Genetic Diagnosis Using Whole Exome Sequencing in Common Variable Immunodeficiency
Source: Front Immunol. 2016 Jun 13;7:220. doi: 10.3389/fimmu.2016.00220 (PMC4903998; doi:10.3389/fimmu.2016.00220)
Supplement: Supplementary file 1 [file table_1.docx]

**Supplementary Material**

**Genetic Diagnosis Using Whole Exome Sequencing in Common Variable Immunodeficiency**

**Patrick Maffucci*, Charles A Filion*, Bertrand Boisson, Yuval Itan, Lei Shang, Jean-Laurent Casanova and Charlotte Cunningham-Rundles^§^**

**^§^Correspondence:** Charlotte Cunningham-Rundles: charlotte.cunningham-rundles@mssm.edu

**Supplemental Table 1.** 269 Gene Screen Panel

| ACP5 | CD27 | ELANE | IL7R | NCF2 | RNASEH2A | TLR3 |
| --- | --- | --- | --- | --- | --- | --- |
| ADA | CD3D | ERBB2IP | INO80 | NCF4 | RNASEH2C | TMC6 |
| ADAR | CD3E | FADD | IRAK4 | NFAT5 | RNASEL | TMC8 |
| AICDA | CD3G | FAS | IRF3 | NFKB1 | RNF168 | TMEM173 |
| AIRE | CD40 | FASLG | IRF4 | NFKB2 | RNF31 | TNFRSF13B |
| AK2 | CD40LG | FCN3 | IRF7 | NHEJ1 | RORC | TNFRSF13C |
| AP3B1 | CD46 | FERMT3 | IRF8 | NHP2 | RPSA | TNFRSF1A |
| AP3D1 | CD59 | FOXN1 | ISG15 | NLRC4 | RTEL1 | TNFRSF4 |
| APOL1 | CD70 | FOXP3 | ITCH | NLRP12 | SAMHD1 | TNFSF12 |
| ATM | CD79A | G6PC3 | ITGAX | NLRP3 | SBDS | TPP2 |
| B2M | CD79B | G6PD | ITGB2 | NOD2 | SERPING1 | TRAC |
| BCL10 | CD81 | GATA2 | ITK | NOP10 | SH2D1A | TRAF3 |
| BLM | CD8A | GFI1 | JAGN1 | NRAS | SLC11A1 | TRAF3IP2 |
| BLNK | CEBPE | GINS1 | JAK3 | ORAI1 | SLC35C1 | TREX1 |
| BLOC1S6 | CFB | HAX1 | KRAS | PCNA | SMARCAL1 | TTC37 |
| BTK | CFD | IFIH1 | LAMTOR2 | PGM3 | SP110 | TTC7A |
| C1QA | CFH | IFNGR1 | LCK | PIK3CD | SPINK5 | TYK2 |
| C1QB | CFHR1 | IFNGR2 | LIG1 | PIK3R1 | STAT1 | UNC119 |
| C1QC | CFI | IGHM | LIG4 | PLCG2 | STAT2 | UNC13D |
| C1R | CFP | IGKC | LIPA | PMS2 | STAT3 | UNC93B1 |
| C1S | CIB1 | IGLL1 | LPIN2 | PNP | STAT5B | UNG |
| C2 | CIITA | IKBKB | LRBA | POLE | STIM1 | USB1 |
| C3 | CLPB | IKBKG | LYST | PRF1 | STK4 | VPS45 |
| C4A | COLEC11 | IKZF1 | MAGT1 | PRKCD | STX11 | WAS |
| C4B | COPA | IL10 | MALT1 | PRKDC | STXBP2 | WIPF1 |
| C5 | CORO1A | IL10RA | MAP3K14 | PSMB8 | TADA2A | XIAP |
| C6 | CR2 | IL10RB | MASP1 | PSTPIP1 | TAP1 | XRCC4 |
| C7 | CSF2RA | IL12B | MASP2 | PTPN6 | TAP2 | ZAP70 |
| C8A | CSF2RB | IL12RB1 | MBL2 | PTPRC | TAPBP | ZBTB24 |
| C8B | CTLA4 | IL12RB2 | MCM4 | RAB27A | TBK1 |  |
| C8G | CTPS1 | IL17F | MEFV | RAC1 | TBX1 |  |
| C9 | CXCR4 | IL17RA | MKL1 | RAC2 | TTC37 |  |
| CARD11 | CYBA | IL17RC | MOGS | RAG1 | TCF3 |  |
| CARD14 | CYBB | IL18 | MPO | RAG2 | TECR |  |
| CARD9 | DCLRE1B | IL1RN | MRE11A | RBCK1 | TERC |  |
| CASP10 | DCLRE1C | IL21 | MSN | RFX5 | TERT |  |
| CASP8 | DKC1 | IL21R | MVK | RFXANK | TGFBR1 |  |
| CCBE1 | DNMT3B | IL2RA | MYD88 | RFXAP | TGFBR2 |  |
| CD19 | DOCK2 | IL2RG | NBN | RHOH | TICAM1 |  |
| CD247 | DOCK8 | IL36RN | NCF1 | RMRP | TINF2 |  |
