# Supplementary material for: Genetic Diagnosis Using Whole Exome Sequencing in Common Variable Immunodeficiency
Source: Front Immunol. 2016 Jun 13;7:220. doi: 10.3389/fimmu.2016.00220 (PMC4903998; doi:10.3389/fimmu.2016.00220)
Supplement: Supplementary file 2 [file table_2.docx]

**Supplementary Material**

**Genetic Diagnosis Using Whole Exome Sequencing in Common Variable Immunodeficiency**

**Patrick Maffucci*, Charles A Filion*, Bertrand Boisson, Yuval Itan, Lei Shang, Jean-Laurent Casanova and Charlotte Cunningham-Rundles^§^**

**^§^Correspondence:** Charlotte Cunningham-Rundles: charlotte.cunningham-rundles@mssm.edu

**Supplemental Table 2.** Sequencing Primers

| **Gene** | **Variation(s)** | **Forward Primer (5’🡪3’)** | **Reverse Primer (5’🡪3’)** |
| --- | --- | --- | --- |
| **CTLA4** | c.56_57insCTGG | ACGTAACAGCTAAACCCACGG | CAGCCAAGCCAGATTGGAGTT |
|  | c.406C>G | AGAGGGGAAGGGGTAAGTGA | ATGCTATTAAGGTTGCCACCCA |
| **DOCK8** | c.3023G>A | AAGAGGACACCCTTCCCACC | ATTTTCCAGCCCCACATCAGG |
|  | c.3312G>C | ACCATCTGTTGCACTATCCC | GTGGTGTAACTTCAGGGTAGC |
| **IKZF1** | c.551G>A | CCCACGCTGAGTTTAGTTCTC | AGCCAGCAAGGACACAATC |
| **LRBA** | c.1399A>G | ATTGTGTTTCTTACAGCTTCCA | CTCCAGGTACTAACTGTGATCTT |
|  | c.2674G>A | TCCTTTATGCTGTGGCAATAGA | GTACCACATTGTCGAATTGTTCTT |
|  | c.6695T>C | CCCTTGCTCTAGAATCATGTAAAC | ACCTCTGTGGCTAGTCTAAATC |
|  | c.8351C>G | TGTAAGCCAACACGACCAG | CACACCATTCCAGTAGCATTTG |
| **NFKB1** | c.259-4A>G | TTAAGTGGCCTGCTTCTTGG | CTGAGGGTCTCAGAAAGGTCC |
|  | c.957T>A | AGCTTCGTAGTACATTAGCATAGC | AGTAGAGGAAAGGTTTTGGTTCAC |
|  | c.1301-1G>A & c.1375delT | GTCTCTTACCTTTATCTGACCTAC | CTGCATGATTATGACTGGAG |
| **PIK3CD** | c.3061G>A | CATTTCTCCAGTAGGGGAGGC | GGCGTTTCCGTTTATGGCTG |
| **PRF1** | c.853_855delAAG | CCCAGGCTGAGTACTGCTCG | TTTGATTGAATGGGGGAAATACTCC |
| **RAG1** | c.577G>A | GGCAAAGCGATCCATCAAGC | GGGAAGTCCACTGCAAGGAG |
| **RAG2** | c.644C>T | GGGTCCAATCTGGGGTCTCC | AGGACGCTCATACATGCCTTC |
| **STAT3** | c.307C>T | AGTTGTTTGATTTTCCATTCCTCC | CCATGGTCTGCTGCTGATTTTT |
|  | c.737G>A | TCTGTGGGCCTGCAGTTAAGA | GTTCCTGCTCTGGAGTTGACTAA |
|  | c.937T>C | CCATGACCAGAAGTCAGCCC | TCCTTCCCCTTCTCCATCTCA |
| **STXBP2** | c.474_483delinsGA | GGGGAGGTACCCACAGAGTC | CATCAGGGGATGGGGTCAAG |
|  | c.1001C>T | TCCTGAGGACCTTCTGTGAG | ACAAAGGCTGAGTCAAGGCAG |
