# Supplementary material for: Genetic Diagnosis Using Whole Exome Sequencing in Common Variable Immunodeficiency
Source: Front Immunol. 2016 Jun 13;7:220. doi: 10.3389/fimmu.2016.00220 (PMC4903998; doi:10.3389/fimmu.2016.00220)
Supplement: Supplementary file 3 [file table_3.docx]

**Supplementary Material**

**Genetic Diagnosis Using Whole Exome Sequencing in Common Variable Immunodeficiency**

**Patrick Maffucci*, Charles A Filion*, Bertrand Boisson, Yuval Itan, Lei Shang, Jean-Laurent Casanova and Charlotte Cunningham-Rundles^§^**

**^§^Correspondence:** Charlotte Cunningham-Rundles: charlotte.cunningham-rundles@mssm.edu

**Supplemental Table 3.** *TNFRSF13B* Mutations^a^

| **Patient** | **Gene** | **Refseq Transcript** | **Coding Change** | **Protein Change** | **CADD** | **ExAC Freq** |
| --- | --- | --- | --- | --- | --- | --- |
| **11** | *TNFRSF13B* | NM_012452.2 | c.37A>G | p.S13G | 0.062 | - |
| **16** |  |  | c.204dupA | p.L69Tfs*12^b (ref 14)^ | 28.6 | 0.0003885 |
| **17** |  |  | c.310T>C | p.C104R^b (ref 14)^ | 25.9 | 0.003212 |
| **18** |  |  | c.310T>C | p.C104R^b (ref 14)^ | 25.9 | 0.003212 |
| **19** |  |  | c.310T>C | p.C104R^b (ref 14)^ | 25.9 | 0.003212 |
| **20** |  |  | c.310T>C | p.C104R^b (ref 14)^ | 25.9 | 0.003212 |
|  |  |  | c.581_582delinsAA | p.S194X^b (ref 14)^ | 35 | 0.000008257^c^ |
| **21** |  |  | c.542C>A | p.A181E^b (ref 14)^ | 22.8 | 0.005360 |
| **22** |  |  | c.542C>A | p.A181E^b (ref 14)^ | 22.8 | 0.005360 |

*^a^*The *TNFRSF13B* variations in this table are all mono-allelic with the exception of patient 20, whose variations are bi-allelic.

^b^Published disease-associated variant. ^c^Variant reported in a single patient in the ExAC database.
