# Supplementary material for: Genetic Diagnosis Using Whole Exome Sequencing in Common Variable Immunodeficiency
Source: Front Immunol. 2016 Jun 13;7:220. doi: 10.3389/fimmu.2016.00220 (PMC4903998; doi:10.3389/fimmu.2016.00220)
Supplement: Supplementary file 4 [file table_4.docx]

**Supplementary Material**

**Genetic Diagnosis Using Whole Exome Sequencing in Common Variable Immunodeficiency**

**Patrick Maffucci*, Charles A Filion*, Bertrand Boisson, Yuval Itan, Lei Shang, Jean-Laurent Casanova and Charlotte Cunningham-Rundles^§^**

**^§^Correspondence:** Charlotte Cunningham-Rundles: charlotte.cunningham-rundles@mssm.edu

**Supplemental Table 4.** Clinical and Immunologic Phenotypes of Patients *TNFRSF13B (TACI)* mutations

| **Patient** | **Sex** | **Year born/**  **Age at onset (years)** | **Infections** | **Other Conditions** | **IgG**  **(7.00-16.00 g/L)^a^** | **IgA**  **(0.70-4.00**  **g/L)^a^** | **IgM**  **(0.40-2.30**  **g/L)^a^** | **CD3+**  **(750-2500/mm^3^)^a^** | **CD4+**  **(480-1700/mm^3^)^a^** | **CD8+**  **(180-1000/mm^3^)^a^** | **CD3-CD56+**  **(135-525/ mm^3^)^a^** | **CD19+**  **(75-375/mm^3^)^a^/**  **CD19+CD27+IgD-%^b^** |
| --- | --- | --- | --- | --- | --- | --- | --- | --- | --- | --- | --- | --- |
| 11 | F | 2000/  3 | Chronic otitis media; Pneumonias; Sinusitis; Periorbital cellulitis; HSV infection; Conjunctivitis; Giardiasis | Chronic colitis with malabsorption and short stature; ILD;  Nodular hyperplasia; Osteoporosis; Splenomegaly | 5.84 (↓) | <0.11 (↓) | 5.87 (↑) | 1033 | 278 (↓) | 594 | 186 | 388/0.65 |
| 16^c^ | M | 1992/  17 | Recurrent otitis media;  Chronic giardiasis;  Chronic Norovirus infection;  Mycoplasma arthritis | Malabsorption and weight loss; Osteoporosis | 0.62 (↓) | <0.05 (↓) | 0.05 (↓) | 549 (↓) | 320 (↓) | 190 | 74 (↓) | 150/0.09 |
| 17 | M | 1998/  3 | Recurrent sinusitis | Enteropathy; Macrocytosis;  Failure to thrive | 0.50 (↓) | <0.05 (↓) | <0.05 (↓) | NA | NA | NA | NA | 1.72%^d^ (↓)/2.79 |
| 18 | M | 1963/  18  Died at 48 | Pneumonia; Recurrent sinusitis (s/p surgery); Giardiasis;  *C. difficile* colitis; | Pulmonary nodules; ITP; Gastric plasmablastic lymphoma | 1.53 (↓) | <0.05 (↓) | 0.15 (↓) | NA | NA | NA | NA | 0.77%^d^ (↓) |
| 19 | F | 1976/  27 | Frequent bronchitis and otitis media in childhood; Recurrent pneumonias; Giardiasis; Viral meningitis | Pulmonary granulomas; Nodular regenerative hyperplasia;  LIP (treated with rituximab);  Splenomegaly with hypersplenism (s/p splenectomy) | Tx^e^ | <0.05 (↓) | <0.05 (↓) | 1476 | 886 | 601 | 329 | 136 (↓) |
| 20 | F | 1962/  16 | Bronchitis;  Giardiasis | Nodular regenerative hyperplasia with cirrhosis, portal hypertension and ascites; ILD with pulmonary hypertension; ITP; AIHA; Superior mesenteric vein thrombosis | 2.10 (↓) | <0.07 (↓) | 0.62 | 5157 | 2294 | 2247 | 426 | 2 (↓)/3.27 |
| 21 | F | 1981/  10 | Pneumonias;  Bronchitis;  Chronic sinusitis | Enteropathy; Pulmonary granulomas; ITP (s/p splenectomy); Uveitis; Arthritis; Skin and eye granulomas | 4.66 (↓) | <0.07 (↓) | 0.34 (↓) | NA | NA | NA | NA | 30.80%^d^/0.08 |
| 22 | F | 1991/  12 | Recurrent otitis media requiring myringotomy tubes;  Sinusitis |  | 3.71 (↓) | 0.14 (↓) | 0.04 (↓) | NA | NA | NA | NA | 24.0%^d^/6.2 |

AIHA = Autoimmune hemolytic anemia; HSV = Herpes simplex virus; ILD = Interstitial lung disease; ITP = Immune thrombocytopenic purpura; LIP = Lymphocytic interstitial pneumonia; NA = Not available

^a^Normal value ranges in patients aged six or more. ^b^Percentage of total CD19+ cells. ^c^Brother of patient 33. ^d^Absolute count was not available. ^e^Patients were already on IgG replacement therapy when evaluated for the first time in our center.
