# Supplementary material for: Genetic Diagnosis Using Whole Exome Sequencing in Common Variable Immunodeficiency
Source: Front Immunol. 2016 Jun 13;7:220. doi: 10.3389/fimmu.2016.00220 (PMC4903998; doi:10.3389/fimmu.2016.00220)
Supplement: Supplementary file 5 [file table_5.docx]

**Supplementary Material**

**Genetic Diagnosis Using Whole Exome Sequencing in Common Variable Immunodeficiency**

**Patrick Maffucci*, Charles A Filion*, Bertrand Boisson, Yuval Itan, Lei Shang, Jean-Laurent Casanova and Charlotte Cunningham-Rundles^§^**

**^§^Correspondence:** Charlotte Cunningham-Rundles: charlotte.cunningham-rundles@mssm.edu

**Supplemental Table 5.** Other Mutations

| **Patient** | **Gene** | **Refseq Transcript** | **Coding Change** | **Protein Change** | **CADD** | **ExAC Freq** |
| --- | --- | --- | --- | --- | --- | --- |
| **23** | *AP3D1* | NM_003938.6 | c.398C>T | p.T133M | 25.3 | 0.00001662 |
| **24** | *C5* | NM_001735.2 | c.4489G>A | p.E1497K | 35 | 0.00001648 |
| **19** | *C6* | NM_000065.3 | c.2461G>A | p.E821K | 15.83 | 0.000008246^a^ |
| **3** | *CIITA* | NM_000246.3 | c.2063G>A | p.W688*^b (ref 28)^ | 37 | - |
| **13** | *DCLRE1C* | NM_001033855.2 | c.212C>T | p.T71M | 23.8 | 0.0001663 |
| **25** | *DCLRE1C* | NM_001033855.2 | c.1334G>A | p.R445H | 4.006 | 0.0002472 |
| **26** | *DCLRE1C* | NM_001033855.2 | c.1464G>T | p.Q488H | 22.9 | 0.00001650 |
| **17** | *DNMT3B* | NM_006892.3 | c.167C>T | p.S56F | 24.7 | 0.00001647 |
| **19** | *DOCK8* | NM_203447.3 | c.3023G>A | p.R1008Q | 35 | 0.0008073 |
|  |  |  | c.3312G>C | p.E1104D | 23.4 | 0.0007838 |
| **18** | *FAS* | NM_000043.4 | c.950A>G | p.D317G | 23.7 | 0.000008318^a^ |
| **27** | *FASLG* | NM_000639.2 | c.592C>T | p.R198W | 32 | - |
| **28** | *FASLG* | NM_000639.2 | c.592C>T | p.R198W | 32 | - |
| **29** | *HAX1* | NM_006118.3 | c.102_103insGAGGAA | p.D34delinsDEE | 16.11 | 0.00001651 |
| **7** | *IL12RB1* | NM_005535.2 | c.320T>C | p.V107A | 19 | 0.00009903 |
| **30** | *IRF3* | NM_001571.5 | c.87G>C | p.K29N | 20.6 | 0.00003310 |
| **4** | *IRF7* | NM_001572.3 | c.1259G>A | p.R420Q | 15.96 | - |
| **15** | *IRF7* | NM_001572.3 | c.1387G>A | p.E463K | 25.8 | - |
| **31** | *LIG4* | NM_002312.3 | c.95G>T | p.R32L | 25.2 | 0.00001666 |
| **24** | *LRBA* | NM_006726.4 | c.3914G>A | p.R1305H | 34 | 0.00007419 |
| **32** | *LRBA* | NM_006726.4 | c.3914G>A | p.R1305H | 34 | 0.00007419 |
| **33** | *LRBA* | NM_006726.4 | c.3914G>A | p.R1305H | 34 | 0.00007419 |
| **2** | *LRBA* | NM_006726.4 | c.4334G>A | p.R1445Q^b (ref 20)^ | 34 | 0.000008301^a^ |
| **26** | *MKL1* | NM_020831.4 | c.413C>T | p.P138L | 26.9 | 0.000008240^a^ |
| **2** | *NCF2* | NM_000433.3 | c.1384G>A | p.V462M | 29.3 | - |
| **20** | *NFAT5* | NM_001113178 | c.92C>A | p.P31Q | 23.3 | 0.00005061 |
| **21** | *NLRC4* | NM_001199139.1 | c.3033T>G | p.D1011E | 23.2 | 0.00004997 |
| **7** | *ORAI1* | NM_032790.3 | c.79_80insGCCGCCGGAGCCGCC | p.S27delinsSRRSRR | 13.68 | - |
| **16** | *PGM3* | NM_015599.2 | c.468G>T | p.L156F | 22.6 | - |
| **15** | *PGM3* | NM_015599.2 | c.1085A>T | c.E362V | 29.2 | - |
| **21** | *PLCG2* | NM_002661.4 | c.77C>T | p.T26M | 24.3 | 0.0006045 |
| **34** | *PLCG2* | NM_002661.4 | c.2631G>C | p.E877D | 15.87 | 0.0001243 |
| **19** | *PMS2* | NM_000535.5 | c.1379G>A | p.G460D | 2.648 | - |
| **35** | *POLE* | NM_006231.3 | c.3491C>T | p.P1164L | 34 | - |
| **36** | *POLE* | NM_006231.3 | c.4522C>G | p.R1508G | 19.8 | - |
| **37** | *POLE* | NM_006231.3 | c.6301G>T | p.A2101S | 24.1 | - |
| **15** | *PRF1* | NM_001083116.1 | c.853_855delAAG | p.K285del^b,c^ | 0.942 | 0.00005766 |
| **26** | *RAG1*^d^ | NM_000488.2 | c. 577G>A | p.E193K | 21.8 | 0.004120 |
|  | *RAG2*^d^ | NM_000536.3 | c. 644C>T | p.T215I^b,e^ | 21.4 | 0.003576 |
| **19** | *SERPING1* | NM_000062.2 | c.850G>A | p.D284N | 22.6 | - |
| **36** | *SERPING1* | NM_000062.2 | c.935C>G | p.P312R | 1.296 | 0.000008237^a^ |
| **28** | *SPINK5* | NM_006846.3 | c.2276G>A | p.R759H | 4.93 | 0.00007460 |
| **4** | *STXBP2* | NM_006949.3 | c.1468C>G | p.R490G | 24.1 | 0.000008801^a^ |
| **35** | *TMC8* | NM_152468.4 | c.1360G>T | p.D454Y | 25 | 0.00004987 |
| **38** | *TRAF3IP2* | NM_147686.3 | c.968A>G | p.H323R | 13.69 | - |

^a^Variant reported in a single patient in the ExAC database. ^b^Published disease-causing variant. ^c^Described by Göransdotter, *et al.* Am J Hum Genet 2001 and by Chia, *et al.* Blood 2012. ^d^Patient’s mother was also found to have both mutations in RAG1 and RAG2 by Sanger sequencing. ^e^Described by Tabori, *et al.* Clin Genet 2004 and by Meshaal, *et al*. Clin Immunol 2015.
