# Supplementary material for: Genetic Diagnosis Using Whole Exome Sequencing in Common Variable Immunodeficiency
Source: Front Immunol. 2016 Jun 13;7:220. doi: 10.3389/fimmu.2016.00220 (PMC4903998; doi:10.3389/fimmu.2016.00220)
Supplement: Supplementary file 6 [file table_6.docx]

**Supplementary Material**

**Genetic Diagnosis Using Whole Exome Sequencing in Common Variable Immunodeficiency**

**Patrick Maffucci*, Charles A Filion*, Bertrand Boisson, Yuval Itan, Lei Shang, Jean-Laurent Casanova and Charlotte Cunningham-Rundles^§^**

**^§^Correspondence:** Charlotte Cunningham-Rundles: charlotte.cunningham-rundles@mssm.edu

**Supplemental Table 6.** Immunological and Clinical Phenotypes of Patients with Other Damaging Mutations

| **Pt** | **Genes** | **Sex** | **Age/**  **Age at onset (years)** | **Infections** | **Other Conditions** | **IgG**  **(700-1600 g/L)^a^** | **IgA**  **(70-400**  **g/L)^a^** | **IgM**  **(40-230**  **g/L)^a^** | **CD3+**  **(750-2500/mm^3^)^a^** | **CD4+**  **(480-1700/mm^3^)^a^** | **CD8+**  **(180-1000/mm^3^)^a^** | **CD3-CD56+ (135-525/ mm^3^)^a^** | **CD19+**  **(75-375/mm^3^)^a^/**  **CD19+CD27+IgD-%^b^** |
| --- | --- | --- | --- | --- | --- | --- | --- | --- | --- | --- | --- | --- | --- |
| 2^c^ | *LRBA*  *NCF2* | F | 33/  19 | Chronic sinusitis; Pneumonias; Conjunctivitis; Shingles;  Otitis; *C. difficile* colitis | Morphea | <0.51 (↓) | <0.05 (↓) | <0.05 (↓) | 1428 | 1007 | 435 | 53 (↓) | 21 (↓)/NA |
| 3 | *CIITA* | M | 48/  21 | Pneumonias; Empyema (s/p lobectomy); Chronic sinusitis | Bronchiectasis  Vitiligo, Hypothyroidism | Tx^d^ | <0.01 | <0.05 (↓) | 682 (↓) | 323 (↓) | 336 | 45 (↓) | 62 (↓)/0 |
| 4 | *IRF7*  *STXBP2* | F | Died at 48/  19 | MAI; *Pneumocystis jirovecii* Pneumonia;  Lung abcesses; PML (cause of death) | ITP (s/p splenectomy);  AIHA;  Aplastic bone marrow | Tx^d^ | 0.07 (↓) | 0.17 (↓) | NA | 1177 | 589 | NA | 0 (↓)/0 |
| 7 | *IL12RB1*  *ORAI1* | M | 18/  3 | *Molluscum contagiosum*;  Staphylococcus infections; Tinea corporis; *C. difficile* colitis | Type 1 diabetes;  ITP/Evans syndrome Hypothyroidism; Short stature | 6.81 (↓) | <0.07 (↓) | 0.37 | 715 (↓) | 337 (↓) | 276 | 83 (↓) | 401/NA |
| 13 | *DCLRE1C* | F | Died at 39/  25 | Cellulitis; Pneumonias | Liver and pulmonary granulomas leading to organ failures (cause of death); ITP; Splenectomy | 5.56 (↓) | <0.07 (↓) | 0.17 (↓) | 2995 | 1579 | 1479 | 363 | 4(↓)/0 |
| 15 | *IRF7*  *PGM3*  *PRF1* | M | 35/  28 | EBV age 28 with neutropenia;  Recurrence of EBV at age 34;  Recurrent sinusitis;  Shingles x2 | IBD | 0.81 (↓) | <0.05 (↓) | <0.05 (↓) | 1623 | 1250 | 304 | 32 (↓) | 9 (↓)/0 |
| 16^e^ | *PGM3* | M | 1992/  17 | Recurrent otitis media;  Chronic giardiasis;  Chronic Norovirus infection;  Mycoplasma arthritis | Malabsorption and weight loss; Osteoporosis | 0.62 (↓) | <0.05 (↓) | 0.05 (↓) | 549 (↓) | 320 (↓) | 190 | 74 (↓) | 150/0.09 |
| 17 | *DNMT3B* | M | 1998/  3 | Recurrent sinusitis | Enteropathy; Macrocytosis;  Failure to thrive | 0.50 (↓) | <0.05 (↓) | <0.05 (↓) | NA | NA | NA | NA | 1.72%^f^ (↓)/2.79 |
| 18 | *FAS* | M | 1963/  18  Died at 48 | Pneumonia; Recurrent sinusitis (s/p surgery); Giardiasis;  *C. difficile* colitis; | Pulmonary nodules; ITP; Gastric plasmablastic lymphoma | 1.53 (↓) | <0.05 (↓) | 0.15 (↓) | NA | NA | NA | NA | 0.77%^f^ (↓) |
| 19 | *C6*  *DOCK8*  *PMS2*  *SERPING1* | F | 1976/  27 | Frequent bronchitis and otitis media in childhood; Recurrent pneumonias; Giardiasis; Viral meningitis | Pulmonary granulomas; Nodular regenerative hyperplasia;  LIP (treated with rituximab);  Splenomegaly with hypersplenism (s/p splenectomy) | Tx^d^ | <0.05 (↓) | <0.05 (↓) | 1476 | 886 | 601 | 329 | 136 (↓) |
| 20 | *NFAT5* | F | 1962/  16 | Bronchitis;  Giardiasis | Nodular regenerative hyperplasia with cirrhosis, portal hypertension and ascites; ILD with pulmonary hypertension; ITP; AIHA; Superior mesenteric vein thrombosis | 2.10 (↓) | <0.07 (↓) | 0.62 | 5157 | 2294 | 2247 | 426 | 2 (↓)/3.27 |
| 21 | *NLRC4*  *PLCG2* | F | 1981/  10 | Pneumonias;  Bronchitis;  Chronic sinusitis | Enteropathy; Pulmonary granulomas; ITP (s/p splenectomy); Uveitis; Arthritis; Skin and eye granulomas | 4.66 (↓) | <0.07 (↓) | 0.34 (↓) | NA | NA | NA | NA | 30.80%^f^/0.08 |
| 23 | *AP3D1* | M | 1957/  33 | *Candida dubliniensis* and *Candida glabrata* pneumonia | Bronchiectasis; Diabetes mellitus; Kleinfelter syndrome; Factor V Leiden deficiency;  DVT; COPD | 3.56 (↓) | <0.05 (↓) | <0.05 (↓) | 354 (↓) | 269 (↓) | 76 (↓) | 71 (↓) | 52 (↓)/0 |
| 24 | *C5*  *LRBA* | M | 1978/  10 | Recurrent sinusitis; Salmonellosis; Conjunctivitis | Nodular regenerative hyperplasia with portal hypertension;  Follicular bronchiolitis; LIP;  Multiple episodes of AIHA; ITP; Lymphoid hyperplasia; Splenomegaly | 4.83 (↓) | 0.06 (↓) | 0.26 | 690 (↓) | 569 | 103 | 225 | 72 (↓)/0.09 |
| 25 | *DCLRE1C* | F | 2005/  1 | Recurrent pneumonias;  Recurrent otitis media, Pharyngitis and bronchitis; | Severe ITP;  Splenomegaly; Lymphoid hyperplasia | 2.15 (↓) | <0.01 (↓) | 0.74 | 1947 | 1327 | 535 | 180 | 418/0.04 |
| 26 | *DCLRE1C*  *MKL1*  *RAG1*  *RAG2* | M | 1986/  12 | Recurrent pneumonia;  Salmonellosis;  Leg ulceration; *Campylobacter jejuni* sepsis |  | Tx^d^ | <0.05 (↓) | <0.05 (↓) | 763 | 288 (↓) | 452 | 15 (↓) | 0 (↓)/0 |
| 27^g^ | *FASLG* | F | 1950/6 | Recurrent pneumonias, bronchitis and sinusitis | COPD; Pericarditis | 1.36 (↓) | <0.15 (↓) | 0.05 (↓) | NA | NA | NA | NA | 1.30%^f^ (↓)/3.81 |
| 28^g^ | *FASLG*  *SPINK5* | F | 1937/6 | Recurrent pneumonias | COPD; Peptidic ulcers; Colitis; Osteoporosis | 573 (↓) | 14 (↓) | 12 (↓) | NA | NA | NA | NA | NA |
| 29 | *HAX1* | M | 1988/  20 | Pneumonias; Bronchitis; Cellulitis leading to *Strepcoccus bovis* sepsis | Pulmonary granulomas;  AIHA; ITP;  Splenomegaly; Lymphadenopathy | 3.45 | 0.00 (↓) | 0.16 (↓) | 166 (↓) | 137 (↓) | 25 (↓) | 18 (↓) | 1 (↓)/NA |
| 30 | *IRF3* | F | 1949/  20 | Recurrent sinopulmonary infections | IBD; ITP; Iritis/Uveitis; Splenomegaly;  Lymphoid hyperplasia | 1.20 | 0.00 | 0.00 | 540 | 279 | 235 | 43 (↓) | 20(↓)/0 |
| 31 | *LIG4* | F | 1964/  13  Died at the age of 47 | Recurrent pneumonias and otitis media | IBD with malabsorption;  Hepatocellular steatosis; Rheumatoid arthritis; MALT lymphoma of the lung;  Meningioma; Bone marrow granulomas;  Pancytopenia;  Brain hemorrhage (cause of death) | 1.80 (↓) | 0.08 (↓) | 0.19 (↓) | 382 (↓) | 202 (↓) | 186 | 15 (↓) | 0 (↓)/0 |
| 32 | *LRBA* | F | 1962/5 | Recurrent pneumonias  Recurrent bronchitis;  Osteomyelitis  Shingles;  Warts on feet and hands | Nodular regenerative hyperplasia; Bronchiectasis;  Pulmonary nodules;  ITP; Lymphoid hyperplasia;  Splenomegaly with hypersplenism | 0.30 (↓) | 0.06 (↓) | 0.07 (↓) | 803 | 590 | 197 | 68 (↓) | 103 (↓)/0.84 |
| 33^e^ | *LRBA* | M | 1998/  1 | Recurrent otitis media;  Giardiasis |  | 1.59 (↓) | <0.05 (↓) | 0.19 | 2251 | 1168 | 953 | 84 (↓) | 335/0.41 |
| 34 | *PLCG2* | M | 2002/  3 | *H. influenzae* and Pseudomonas pneumonias;  Chronic otitis media (s/p tympanectomy);  Kidney infection | Bronchiectasis; Factor V Leiden deficiency | 0.80 (↓) | 0.14 (↓) | <0.05 (↓) | 1412 | 836 | 471 | 76 (↓) | 390/0 |
| 35 | *POLE*  *TMC8* | F | 1999/3 | Chronic sinusitis;  Giardiasis | Liver and pulmonary granulomas; Short stature;  Cyclic neutropenia | 1.39 (↓) | 0.07 (↓) | 0.07 (↓) | 1780 | 842 | 843 | 9 (↓) | 0 (↓)/0 |
| 36 | *POLE*  *SERPING1* | M | 1986/  5 | Pneumonias;  Frequent otitis with hearing loss | ILD; AIHA; Lymphoid hyperplasia;  Splenomegaly; Growth delay/short stature | 3.95 (↓) | <0.07 (↓) | <0.04 (↓) | 288 (↓) | 178 (↓) | 104 (↓) | 58 (↓) | 0 (↓)/0 |
| 37 | *POLE* | F | 1960/  29 | Frequent pneumonias;  Recurrent sinusitis and otitis media | Felty’s syndrome (s/p splenectomy);  Arthritis; Cardiac tamponnade | 9.57^h^ | 0.19 (↓) | 1.64 | 1537 | 782 | 741 | 30 (↓) | 3 (↓)/2.7 |
| 38 | *TRAF3IP2* | M | 1994/  17 | Recurrent MRSA skin infections;  Shingles | Granulomatous ILD; Cervical and axillary granulomas | 2.30 (↓) | 0.08 (↓) | 0.08 (↓) | 696 (↓) | 330 (↓) | 338 | 22 (↓) | 6 (↓)/1.53 |

AIHA = Autoimmune hemolytic anemia; COPD = Chronic obstructive Pulmonary disease; DVT = Deep venous thrombosis; EBV = Epstein-Barr virus; IBD = Inflammatory bowel disease; ILD = Interstitial lung disease; ITP = Immune thrombocytopenic purpura; LIP = Lymphocytic interstitial pneumonia; MAI = *Mycobacterium avium intracellulare*; NA = Not available; PML = Progressive multifocal leukoencephalopathy.

^a^Normal value ranges in patients aged six or more. ^b^Percentage of total CD19+ cells. ^c^Second cousin of patient 1. ^d^Patients were already on IgG replacement therapy when evaluated for the first time in our center. ^e^Patients 16 and 33 are brothers. ^f^Absolute count was not available. ^g^Patients 27 and 28 are sisters. ^h^IgG2 and IgG4 were decreased in this patient.
